# Protocol for the derivation, culturing, and differentiation of human iPS-cell-derived neuroepithelial stem cells to study neural differentiation in vitro

**DOI:** 10.1016/j.xpro.2021.100528

**Published:** 2021-05-06

**Authors:** Javier Calvo-Garrido, Dania Winn, Camilla Maffezzini, Anna Wedell, Christoph Freyer, Anna Falk, Anna Wredenberg

**Affiliations:** 1 Department of Medical Biochemistry and Biophysics, Karolinska Institutet, Stockholm, 171 77, Sweden; 2 Department of Neuroscience, Karolinska Institutet, Stockholm, 171 77, Sweden; 3 Department of Molecular Medicine and Surgery, Karolinska Institutet, Stockholm, 171 77, Sweden; 4 Centre for Inherited Metabolic Diseases, Karolinska University Hospital, Stockholm, 171 65, Sweden; 5 Current address: Stem Cell and Neurogenesis Unit, Division of Neuroscience, San Raffaele Scientific Institute, 20132, Milan, Italy

**Keywords:** Neuroscience, Stem Cells, Cell Differentiation

## Abstract

Here, we present a revised protocol to derive neuroepithelial stem (NES) cells from human induced pluripotent stem cells. NES cells can be further differentiated into a culture of neurons (90%) and glia (10%). We describe how to derive and maintain NES cells in culture and how to differentiate them. In addition, we show the potential use of NES cells to study the role of reactive oxygen species in neuronal differentiation and a guideline for NES cell transfection.

For complete details on the use and execution of this protocol, please refer to Calvo-Garrido et al. (2019); Falk et al. (2012).

## Highlights

• NES cells, neurons, and glia generated through dual SMAD inhibition
• Proliferate, maintain, and differentiate NES cells into neurons and glia in culture
• Coating of the growth surface, cell density, and trypsinization need to be adjusted
• Feasible model to study ROS effect in neuronal and glia differentiation

## Graphical abstract

**Figure:**
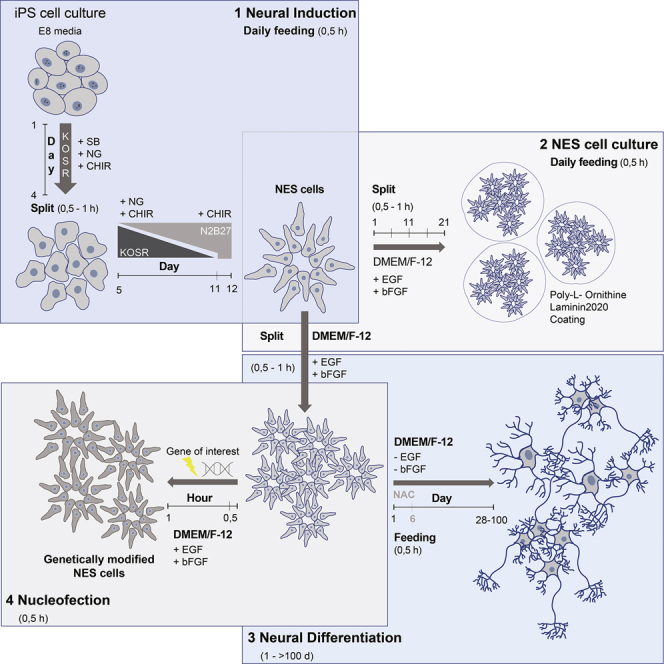

## Before you begin

### Reagent and factor preparation for iPS cell culture

**Timing: 15 min**

1. Aliquot laminin521 and E8 supplement to avoid repeated freezing-thaw cycles.

### Preparation of stock solutions for NES cell derivation, culture, and differentiation

**Timing: 1 h**

Prepare aliquots of stock solutions to reduce freeze-thawing cycles. See Table 2 for storage and working concentrations.

2. Resuspend and filter Poly-L-Ornithine in PBS to a final concentration of 100 μg/mL and aliquot.
3. Resuspend Noggin in PBS at 500 μg/mL and aliquot 20 μL.
4. Knock out serum replacement (KOSR) and non-essential amino acids, aliquot in volumes of 10 mL.
5. Resuspend 1 mg of SB431542 in 260 μL of DMSO to a final concentration of 10 mM and aliquot.
6. Resuspend 1 mg of CHIR99021 in 215 μL of DMSO to a final concentration of 3.3 μM and aliquot.
7. Resuspend bFGF in PBS-BSA 0.15% and EGF in PBS to a final concentration of 100 ng/μL. Aliquot in a volume of 100 μL.
8. Aliquot B27 in 500 μL aliquots and N2 and Penicillin/Streptomycin (P/S) in 5 mL aliquots.
9. **Optional!** Prepare N-Acetyl-L-cysteine (NAC) stock solution at 612 mM. NAC is only necessary if differentiation under modified redox homeostasis is to be investigated. NAC is soluble in dH_2_O after heating at 37°C for five minutes with a concentration of 100 mg/mL or 612 mM (MW 163.19 g/mol). Dilute the stock in dH_2_O for working concentrations of 0.5 mM (low concentration) and 2.5 mM (high concentration).

**Table 1:** Volumes for different cell culture dishes to grow NES cells and coat culture dishes

Culture dish	Growth area (cm^2^)	Volume of coating	Volume of medium
24 well plate	2	0.35 mL	0.5 mL
12 well plate	3.5	0.5 mL	0.6 mL
6 well plate	9.5	1.5 mL	1.5–2 mL
T25 flask	25	4 mL	4–5 mL
T75 flask	75	10 mL	9–10 mL
T-150 flask	150	20 mL	18–20 mL

**Table 2:** Storage and working concentrations of reagents used to culture and differentiate NES cells

Reagent	Storage Concentration	Working Concentration	Storing Conditions
bFGF	100 ng/μL	10 ng/mL	Long term storage at −80°C
EGF	100 ng/μL	10 ng/mL	
B27	Directly aliquoted	1:100 in induction medium 2. 1:1000 in growth medium. 1:100 in differentiation medium.	Store at -20°C
N2	Directly aliquoted	1:200 in induction medium 2. 1:100 in both growth and differentiation media	
hNoggin (NG)	500 μg/mL	500 ng/mL (1:1000)	
SB431542	10 mM	10 μM (1:1000)	
CHIR 99021	10 mM	3.3 μM (1:3000)	
mouse laminin	Directly aliquoted from a 1-2 mg/mL stock	4 μg/mL	
laminin521	Directly aliquoted from a 0.1 mg/mL stock	5 μg/mL	
2-mercaptoethanol	50 mM	90 μM	Store at 4°C
GlutaMAX	Directly aliquoted	1:100	
N-Acetyl-L-cysteine (NAC)	612 mM	0.5 mM/2.5 mM	
KnockOut Serum Replacement (KOSR)	Directly aliquoted	1:5	Store at −20°C (for long term storage)
Poly-L-Ornithine	100 μg/mL	100 μg/mL	
BSA	200 mg/mL	2 mg/mL	
Penicillin-Streptomycin (P/S)	10,000 U/mL	100 U/mL (1:100)	
ROCK inhibitor	5 mM	5 μM (1:1000)	
E8 supplement	Directly aliquoted	1:50	

#### Media for NES cell derivation, culture, and differentiation

**Timing: 1 h**

10. Wash medium
  a. Prepare a 200 mg/mL BSA stock solution in DMEM/F12 media.
  b. Wash medium is prepared by adding 5 mL of BSA stock solution to 500 mL of DMEM/F12 to have a BSA working concentration of 2 mg/mL.
11. Neural induction media
  a. Prepare neural induction medium 1 and 2 according to Tables 3 and 4.

**Table 3:** Composition of neural induction medium-1

KOSR-medium (medium 1)	Volume for 50 mL	Concentration
DMEM/F-12+GlutaMAX	39.5 mL	-
KnockOut Serum Replacement (KOSR)	10 mL	1:5
Non-essential Amino Acids	500 μL	1:100
2-mercaptoethanol	91 μL	1:550
P/S	500 μL	1:100

**Table 4:** Composition of neural induction medium-2

N2B27-medium (medium 2)	Volume for 50 mL	Concentration
DMEM/F-12+GlutaMAX	24 mL	-
Neurobasal	24 mL	-
GlutaMAX	250 μL	1:100 (for Neurobasal)
2-mercaptoethanol	91 μL	1:500
N2	250 μL	1:200
B27	500 μL	1:100
P/S	500 μL	1:100
12. NES growth medium
  a. Prepare NES growth medium according to Table 5.

**Table 5:** Composition of NES growth medium

NES medium	Volume for 50 mL	Concentration
DMEM/F-12+GlutaMAX	48.5 mL	-
N2	500 μL	1:100
B27	50 μL	1:1000
P/S	500 μL	1:100
bFGF	5 μL	1:10 000 (10 ng/mL)
EGF	5 μL	1:10 000 (10 ng/mL)

**Timing: 15 min**

13. NES differentiation medium
  a. Prepare NES differentiation medium according to Table 6.

**Table 6:** Composition of NES differentiation medium

NES differentiation medium	Volume for 50 mL	Concentration
DMEM/F-12+GlutaMAX	49 mL	-
N2	500 μL	1:100
B27	500 μL	1:100
P/S	500 μL	1:100 (optional)

**Table 7:** Volume and working concentrations of reagents used to differentiate NES cells and modulate redox homeostasis

NES differentiation medium+ antioxidant	Volume for 50 mL	Concentration
DMEM/F-12+GlutaMAX	49 mL	-
N2	500 μL	1:100
B27/B27-MA	500 μL	1:100
P/S	500 μL	1:100 (optional)
NAC	40.85 μL (0.5 mM) or 204.25 μL (2.5 mM)	1:1224 (0.5 mM) or 1:244.8 (2.5 mM) of a 0.612 M stock

Neural induction medium 1 and 2 should be prepared fresh every day. NES growth medium, NES differentiation medium and NES differentiation medium + antioxidants can be stored at 4°C for 1 week.
  b. NES differentiation medium consists of DMEM/F12 medium supplemented with N2 (1:100), B27 (1:100). Addition of P/S is optional (1:100). Follow NES cells establishment guidelines for correct storage of factors and medium.
  c. See Table 2 for storage and working concentrations as well as storage conditions of all factors.

### Preparation of stock solutions and media for NES cell nucleofection

**Timing: 1 h**

14. Aliquot buffer P3 from the Lonza nucleofection kit. Lonza offers several buffers to perform the nucleofection in P3 is the most efficient for NES cells.
15. Measure plasmid DNA concentration and prepare the right amount in a small volume so buffer P3 composition is not altered. Try different concentrations of DNA to identify the one working the best for your experimental set-up.
16. NES growth medium
  a. Follow NES cells growth procedure for reagents, factors preparation, and medium preparation.

**CRITICAL:** Avoid repeated freeze-thaw cycles of frozen aliquots of EGF and bFGF. The volume of the aliquots should be determined according to the average volume used during a week.

***Note:*** The reagents are not toxic or harmful to the user and do not require any special handling.

**CRITICAL:** The addition of P/S is not mandatory. We have not observed differences when growing NES cells in the presence or absence of these antibiotics.

**CRITICAL:** NES growth medium or NES differentiation medium can be stored for a maximum of 1 week at +4°C. This is also applicable for NES differentiation medium containing NAC.

### Coating of plastic dishes for iPS cell culture

**Timing: 30 min**

17. Coating of cell culture plates with laminin521 for iPS cell culture
  a. Dilute laminin521 to a working concentration of 5 μg/mL in PBS (we use PBS without Ca^2+^and Mg^2+^, but it should also be possible to use PBS with Ca^2+^and Mg^2+^) and add appropriate volume to each well as indicated in Table 1.
  b. Incubate freshly coated plates at 4°C for at least 12 h. We recommend to seal plates and dishes with parafilm for extended storage (>24 h at +4°C).

### Coating of plastic dishes for freshly established NES cells

**Timing: 1 day**

18. Coat cell culture plates with Poly-L-Ornithine
  a. Resuspend Poly-L-Ornithine in PBS for a working concentration of 100 μg/mL. Add Poly-L Ornithine-PBS solution to the culture plates and incubate for at least 2 h at 37°C (see Table 1 for volumes according to culture dish).
19. Coat cell culture plates with mouse laminin and laminin521
  a. Wash Poly-L-Ornithine coated plates three times with generous amounts of PBS (≥ 1 mL per 3 cm plate).
  b. Dilute mouse laminin (4 mg/mL working concentration) and laminin521 (5 mg/mL working concentration) in PBS. Add appropriate volume of laminin dilution (see Table 1) into each well of the culture plate.
  c. Incubate the plate for at least 12 h at +4°C or until they are needed. Alternatively, incubation at 37°C for at least 2 h is also possible, but coating will be less efficient and is not suitable for prolonged culturing (adequate volumes for different areas are indicated in the materials and equipment section).

### Coating of plastic dishes for established NES cell culture and NES cell differentiation

**Timing: 1 day**

20. Coat cell culture plates with Poly-L-Ornithine
  a. Resuspend Poly-L-Ornithine in PBS for a working concentration of 100 μg/mL. Add appropriate volume of Poly-L Ornithine-PBS solution to each well as indicated in Table 1 and incubate for at least 2 h at 37°C (see Table 1).
21. Coat cell culture plates with mouse laminin
  a. Wash Poly-L-Ornithine coated plates three times with generous amounts of PBS (≥ 1 mL per 3 cm plate).
  b. Dilute mouse laminin to a working concentration of 4 μg/mL in PBS, add appropriate volume to each well as indicated in Table 1 and incubate the plates for at least 12 h at +4°C or until they are needed.
  c. We recommend to seal plates and dishes with parafilm for extended storage (>24 h at +4°C).

### Coating of plastic dishes for nucleofection of NES cells

**Timing: 1 day**

22. Follow NES cell culture guidelines to coat plastic dishes and prepare the number of dishes required to perform the experiment.
23. Prepare the appropriate number of coated culture dishes. When using strips for nucleofection it is recommended to seed each 16-strip well to a single well of a 24 multi-well plate. If cuvettes are used, we recommend using T25 flasks or equivalent plates per cuvette.

**CRITICAL:** Preparation of reagents and medium as well as the cell culture work should be carried out under sterile conditions.

**CRITICAL:** We recommend to seal plates and dishes with parafilm during laminin coating and for extended storage (>24h at +4°C). Nevertheless, dry patches might appear after 1–2 weeks at +4°C. Such plates should be discarded, and we therefore do not recommend coating plastic dishes longer than three weeks in advance.

**CRITICAL:** Coverslips and glass dishes are not optimal surfaces for NES cells culture. For this reason, extra attention should be put to the coating of such surfaces.

**CRITICAL:** Poly-L-Ornithine is toxic for the cells. Thorough washing with generous amounts of PBS prior to laminin treatment is therefore highly recommended.

**CRITICAL:** Mouse laminin batches can vary between 1–2 mg/mL and we therefore recommend a maximum dilution of 1:500 to ensure a sufficient laminin concentration.

**CRITICAL:** Never let dishes dry out. Work only with single (multi-well) dishes or up to three T75 flasks at once. Prevent drying out of the plates after aspiration of Poly-L-Ornithine or during the PBS washes as well as after every step where the complete liquid is removed from the plate.

**CRITICAL:** Aspiration should always be performed by tilting the plate to one side. Never aspirate from the center of the plate.

## Key resources table

**Table:** 

REAGENT or RESOURCE	SOURCE	IDENTIFIER
**Antibodies**		

Rabbit monoclonal anti-β-tubulin III Working dilution, 1:1000	Merck	Cat T2200. RRID:AB_262133
Rabbit monoclonal anti-SOX2 (D6D9) Working dilution, 1:1000	Cell Signaling	Cat #3579 RRID:AB_2195767
Mouse monoclonal anti-Nestin Working dilution, 1:500	Millipore	MAB5326 *RRID*:AB_11211837

**Chemicals, peptides, and recombinant proteins**		

DMEM/F-12, GlutaMAX^TM^ supplement	Thermo Fisher Scientific	31331-028
N-2 Supplement (100**×**)	Thermo Fisher Scientific	17502-048
B27^TM^ Supplement (50**×**), Serum free	Thermo Fisher Scientific	17504-044
B27^TM^ Supplement minus antioxidants (B27-MA ; 50×) Serum free	Thermo Fisher Scientific	10889038
Poly-L-ornithine hydrobromide	Merck	P3665. CAS Number: 27378-49-0
Laminin from Engelbreth-Holm-Swarm murine sarcoma basement membrane	Merck	L2020. CAS Number: 114956-81-9
Soybean Trypsin Inhibitor, powder	Thermo Fisher Scientific	17075-029
Human bFGF Recombinant Protein	Thermo Fisher Scientific	13256-029
Animal-Free Recombinant Human EGF	PeproTech	AF-100-15 Accession number: P01133
N-Acetyl-L-cysteine	Merck	A9165 CAS Number: 616-91-1
Essential 8 Medium Kit (E8) (Basal medium and supplement; 50×)	Thermo Fisher Scientific	A1517001
ROCK Inhibitor (Y-27632)	Merck	SCM075
Human recombinant laminin 521	BioLamina	LN521-02
KnockOut™ Serum Replacement	Thermo Fisher Scientific	10828028
Non-essential Amino Acids 100×	Thermo Fisher Scientific	11140035
2-Mercaptoethanol 50 mM	Thermo Fisher Scientific	31350010
Penicillin-Streptomycin 10,000 U/mL	Thermo Fisher Scientific	15140122
Neurobasal™ Medium	Thermo Fisher Scientific	21103049
TrypLE™ Select Enzyme (1×)	Thermo Fisher Scientific	12563011
Recombinant Human Noggin	PeproTech	120-10C
SB431542	Merck	S4317-5MG
CHIR99021	Merck	SML1046-5MG
DPBS, no calcium, no magnesium	Thermo Fisher Scientific	14190169
BSA	Merck	A7030
GlutaMAX™ Supplement	Thermo Fisher Scientific	35050061

**Experimental models: cell lines**		

Human neuroepithelial stem cells	Falk Lab	N/A
Induced pluripotent stem cells	Falk lab	N/A

**Critical commercial assays**		

Lonza P3 Primary Cell 4D-Nucleofector Kit S or L	Lonza (Fisher Scientific)	V4XP-3024 or -3032 (Lonza) 13429329 or 13439329 (Fisher Scientific)

**Other**		

Plastic dishes	Sarstedt	N/A
Cryo vials	VWR	479-1261P
12-Well plates	VWR	734-2324
4D-Nucleofector	Lonza	AAF-1002B

## Materials and equipment

• All reagents mentioned in Table 2 can be stored in the conditions above for at least 1 year or as long as indicated by the manufacturer.
• All buffers and reagents for the nucleofection can be stored in −20°C.

## Step-by-step method details

### Neural induction – derivation of NES cells from iPS cells

The following section describes how to culture iPSCs using laminin521 coating and E8 media, which is our preferential procedure.

***Note:*** Other culture methods for stable culture of iPSCs are available. Please refer to the appropriate protocols if you wish to pursue them.

#### Culturing iPSCs

**Timing: 4 days**

1. Culture iPS cells until a sufficient cell number for the intended experiment is reached.
  a. Seed 17 500 – 25 000 cells/cm^2^ iPS cells (see Table 8) in a plate coated with laminin521.

**Table 8:** Suggested cell numbers for different cell culture dishes to grow iPS cells at day 0 (D0) and day 5 (D5).

Culture dish	Cell numbers for seeding D0 and for stable NES cells (∗10^6^)	Cell numbers for splitting D5 (∗10^6^)
24 well plate	0.075–0.080	0.380–0.542
12 well plate	0.150–0.200	0.700–1.000
6 well plate	0.375–0.390	1.920–2.736
T25 flask	0.970–0.990	5.000–7.125
  b. Culture iPS cells in complete E8 media containing the E8 supplement (1:50) and P/S (1:100). Split the cells once they reached 80% confluency (after 3–4 days).
  c. Change the media every day.
  d. Add 1:1000 ROCK inhibitor to the media after every splitting and thawing.

**CRITICAL:** Do not warm up the media for more than 15 min at 37°C, but rather keep them outside of the fridge until they reached 21°C and then quickly warm them up in a water bath before using.

**CRITICAL:** All the steps must be performed under sterile conditions.

**CRITICAL:** An appropriate cell density is crucial for a successful induction. Use the recommended cell numbers for seeding (see Table 8).

#### Neural induction

**Timing: 12 days**

2. Prepare KOSR media and N2B27 media according to Tables 3 and 4, respectively.

Day 0

3. Split the iPS cells and seed according to the number of cells in 2-3 wells of your culture dish of choice.
4. Plate the cells in an appropriate volume of complete Essential 8 media (E8 incl. P/S and E8 supplement).
5. Add ROCK inhibitor 1:1000 to the media.

Day 1

Start of neural induction

6. Warm an appropriate volume of KOSR media to 37°C.
7. Prepare the complete KOSR media by adding the factors according to Table 3.
8. Change the media in all wells to KOSR media, according to Table 9.

**Table 9:** Media composition for each day of neural induction

Day	KOSR Media	N2B27 Media	Factors
1–4	100%	0%	NG 1:1000 SB 1:1000 CHIR 1:3000
5–6	75%	25%	NG 1:1000 CHIR 1:3000
7–8	50%	50%	NG 1:1000 CHIR 1:3000
9–10	25%	75%	NG 1:1000 CHIR 1:3000
11–12	0%	100%	CHIR 1:3000

Day 2–4

9. Change the media daily as stated above (see Table 9).

Day 4

10. Coat double the number of wells used before in a fresh culture dish of the same size with laminin521. Keep refrigerated for ≥ 12 h at 4°C.

Day 5

Split cells to a fresh plate

11. Prepare an appropriate volume (see Table 1) of KOSR/N2B27 media (see Tables 3, 4, and 9). Prepare 3 mL extra.
12. Wash the cells once with PBS. Ensure that all PBS is removed after washing.
13. Add 500 μL of TrypLE Express (500 μL are sufficient for culture dishes up to T25) to each well.
14. Incubate at 37°C for maximum 3 min. Rounding and detachment of the cells from the culture surface can be confirmed under a standard light microscope.
15. Meanwhile, prepare a sterile 15 mL tube with an equal volume of KOSR media to TrypLE used.
16. Transfer the detached cells from the culture dish to the 15 mL tube with KOSR media.
17. Centrifuge for 3 min at 300 *g*.
18. Carefully aspirate the media without touching the pellet.
19. Resuspend the cell pellet in 1–2 mL KOSR/N2B27 media and count the cells.
20. Seed the cells with a density of 2–2.85 × 10^5^ cells/cm^2^ and according to Table 8.
21. Add ROCK inhibitor 1:1000 to the media.

Day 6-Day 11

22. Change the media every day according to media composition in Table 9.

Day 11

Coat the necessary plates following NES cell culture section + additional laminin521 for splitting at day 12. Prepare the same type of plate as currently used for the cells. i.e., 12-well plate.

23. Dilute an appropriate volume of poly-L-Ornithine 1:500 in PBS, according to Table 1.
24. Incubate for at least 2 h at 37°C.
25. Remove the Poly-L-Ornithine and wash each well with the volume of PBS stated in Table 1.
26. Dilute mouse laminin for a final concentration of 4 μg/mL and laminin521 for 5 μg/mL in PBS (see Table 2 for working concentrations).
27. Add an appropriate volume (see Table 1) of the diluted laminin solution to each well.
28. Wrap the edges in parafilm and incubate for ≥ 12 h at +4°C.

Day 12

29. Remove the media and wash the cells with PBS.
30. Remove the PBS and add 500 μL of TrypLE to each well.
31. Incubate at 37°C for 2–3 min.
32. Transfer the detached cells from the wells into a tube with the same volume of NES media as the volume of TrypLE.
33. Spin for 3 min at 300 *g*.
34. Aspirate the supernatant carefully and without touching the cell pellet.
35. Resuspend the cells in 1 mL of NES growth medium (Table 5) and count the cells.
36. Plate 1 ∗10^6^ cells per well (12 well plate) in a Poly-L-Ornithine- and laminin (mouse laminin and laminin521) coated 12 well plate.
37. Add ROCK inhibitor 1:1000.

**CRITICAL:** Prepare the media as stated in Tables 3 and 4. Add all factors (see Table 2) fresh every day.

**CRITICAL:** It is essential to pre-warm plastic dishes, TrypLE select, and media to 37°C prior to working with NES cells. For NES growth medium the pre-incubation in a water bath should not exceed 15 min to avoid the degradation or inactivation of included factors.

#### Establishment of NES cells

**Timing: 6 days until the first splitting, after that 3–4 days until the next passage (new NES cell line established after 6 passages)**

See “before you begin” for required stock solutions and media

Day 1–3

38. Wash the newly established NES cells with PBS before changing the media for the first three days after neural induction.
39. Exchange the media daily with fresh NES growth media (see Table 5). See Table 1 for details about the suggested medium volumes.

Day 4–5

40. Change the media every day to fresh NES growth media (see Table 5 for media composition and Table 1 for medium volumes).

Day 6 and after the first passage

41. Split the cells when they reached a confluency of 100%.
42. Add ROCK inhibitor 1:1000 for this passage.
43. Seed the cells onto normal NES cell plates (Poly-L-Ornithine and mouse laminin) (see section “Before You Begin” above.)
44. Passage the cells 1:2 for 5 passages. After this the cells should be seeded with a density of 4 × 10^4^ cells/cm^2^ and without adding ROCK inhibitor to the media.
45. Exchange the media daily with fresh NES growth media (see Table 5 for medium composition and Table 1 for suggested volumes).
46. Monitor the newly established cell lines for the formation of typical NES cell clusters in order to confirm the establishment of NES cells. NES cells will form small rosettes and express NES cell markers, such as NESTIN and SOX2 (Figure 1).

**Figure 1:**
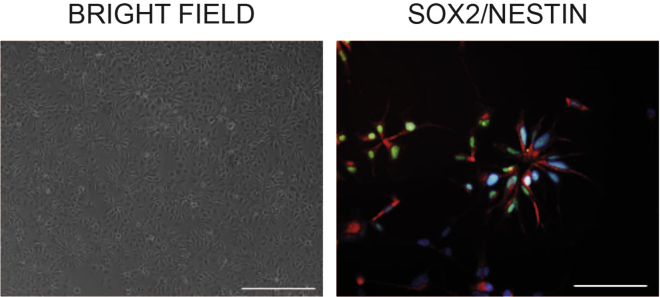
NES cell characteristics (A). Representative bright-field image of a healthy control NES cell line after 7 passages growing in the typical rosette shapes. Scale bar: 200 μm. (B). Immunofluorescence analysis of an established healthy control NES cell line expressing the neural stem cell makers SOX2 (green) and NESTIN (red). Cell nuclei are shown in blue. Scale bar: 100 μm.
47. Freeze several aliquots of the newly established NES cell line. For cryopreservation see the section below.

**CRITICAL:** The right cell density is an important factor for the successful establishment of NES cells. Use the recommended cell numbers for seeding and splitting.

### NES cell culture

#### Thawing of NES cells

**Timing: 30 min**

48. Frozen aliquots of NES cells should be rapidly thawed in a 37°C water bath for not longer than 30 seconds.
49. Add 0.5 mL of warm wash medium dropwise to the vial the cells are in.
50. Transfer the thawed NES cells to a fresh 15 mL sterile tube and add wash medium 5× the volume (see Table 1).
51. Centrifuge the cell suspension for 3 min at 300 *g* and carefully remove the supernatant by pipetting or aspiration.
52. Carefully resuspend the cells in 1 mL of wash media and count them.
53. Calculate how many cells to seed according to the used cell culture plate (see Table 10). Increase the cell number slightly for this step.

**Table 10:** Required number of NES cells to be seeded after splitting or thawing

Culture plate	Growth area (cm^2^)	Seeding number (∗10^6^)
24-MW	2	0.1
12-MW	3.8	0.15
6-MW	9.5	0.5
T-25	25	1
T-75	75	3
T-150	150	6
54. Add an appropriate volume for the planned culture plate of NES growth medium according to Table 1.
55. Remove the laminin solution from the warm tissue culture plates by aspiration and rapidly seed the cells with an appropriate volume of NES growth medium.
56. Replace the NES cell growth medium daily to ensure successful culturing and proliferation.

**CRITICAL:** Always pay attention to all washing steps to remove all residues of DMSO.

**CRITICAL:** Warm the plastic dishes and media before usage. Do not warm complete media in the water bath for more than 15 min.

**CRITICAL:** Increase the number of cells per well after thawing.

#### Maintenance of NES cells

**Timing: daily feeding: 30 min; 3**–**5 days until next splitting**

57. Replace the NES growth medium daily.
58. Split the cells once they reached 100% confluency. This can take between 3–5 days, depending on the seeding density and the cell line.
59. The stated cell numbers in Table 10 are suitable for normal NES cell expansion. For experimental set-ups like immunocytochemistry or electrophysiological recordings cell numbers must be adapted according to the needed cell density. The recommended cell density for NES cell maintenance is 4 × 10^4^ cells/cm^2^.

#### Splitting of NES cells

**Timing: 30 min–1 h**

60. Remove all NES growth medium from the culture dish by slightly tilting the plate to one side. Add an appropriate volume of pre-warmed TrypLE select for 2–3 min to the cells.
61. After incubation gently pipette the cell suspension to detach the remaining cells. If cells remain attached, a slight tapping to the side of the plates or an additional incubation for 1 min at 37°C can be performed.
62. Transfer the cell suspension to a new 15 mL tube containing 5× the volume of wash media and centrifuge for 3 min at 300 *g*.
63. Carefully remove the supernatant by pipetting, aspiration, or decanting, and resuspend the cell pellet in 5 volumes of wash medium (see Table 11).

**Table 11:** Volumes for different cell culture dishes to grow NES cells

Culture dish	Volume of TrypLE select	Volume of wash medium
24 well plate	0.25 mL	1.25 mL
12 well plate	0.5 mL	2.5 mL
6 well plate	0.5 mL	2.5 mL
T25 flask	1 mL	5 mL
T75 flask	2.5 mL	12.5 mL
T-150 flask	5 mL	25 mL
64. Centrifuge the cells for 3 min at 300 *g*.
65. Count the cells.
66. Remove the wash medium as above and carefully resuspend the cells in NES growth medium before seeding the cells at an appropriate concentration on coated plates (Table 10).
67. Aspirate the mouse laminin dilution from the wells before adding the cells.

**CRITICAL:** Excessive pipetting of NES cells and the formation of bubbles should be avoided at all costs. Direct pipetting onto the cell layer should be avoided as much as possible. Similarly, smooth pipetting, when resuspending cell pellets, is advised to prevent cell breakage.

**CRITICAL:** Do not extend trypsinization for longer than three minutes. Excessive trypsinization can cause cell breakage. Disregard residual undetached cells after maximum incubation and proceed with the addition of the trypsin inhibitor medium.

**CRITICAL:** Splitting of confluent NES cell cultures should not exceed a ratio 1:3. Increased dilution ratios can induce neuronal differentiation, repression of the cellular division program and cell death. On the contrary, smaller dilution ratios, such as 1:2, are possible and will stimulate cell division.

**CRITICAL:** It is essential to pre-warm plastic dishes, TrypLE select and all medias to 37°C 30 min prior to working with NES cells. For NES growth medium the incubation in a water bath should not exceed 15 min to avoid degradation or inactivation of included factors.

**CRITICAL:** For NES cell maintenance carefully remove old medium and replace with fresh. Do not add medium directly onto the cells but rather pipet it carefully along the inside of the plate wall.

#### Cryopreservation of NES cells

**Timing: 30 min**

NES cells can be cryopreserved in NES growth medium, supplemented with 10% DMSO (NES freezing medium). Avoid using serum from animal origin. NES cells should be frozen slowly in appropriate freezing containers at −80°C and transferred to the vaporous phase of a liquid nitrogen tank or equivalent 24 h after freezing.

68. Follow the guidelines for splitting of NES cells, but instead of a final resuspension in NES cell growth medium, resuspend the cell pellet in NES freezing medium in a concentration ranging from 2–5 x10^6^ cells/mL and transfer to a cryotube.

**CRITICAL:** Freeze NES cells at concentrations between 2–5 x10^6^ cells/mL.

**CRITICAL:** Storage of frozen cells longer than 24h at −80°C can compromise cell viability.

**CRITICAL:** Work as quick as possible when freezing and thawing the cells so they are not exposed to the cryo media for longer periods of time.

**CRITICAL:** Always pay attention to all washing steps to remove all residues of TrypLE select or DMSO.

**CRITICAL:** Should the storage tank, i.e., a liquid nitrogen tank, be not near the water bath, we recommend transport on dry ice.

### Neural differentiation of NES cells

**Timing: 30 min/day; culture between 14 days-several months**

Change the medium for at least 14 days for a completely developed neuronal morphology. At least 45 days for a mixed culture of neurons and glia. Differentiated cells can be cultures for at least 2 months.

In order to induce neuronal differentiation NES growth medium is modified by removing growth factors bFGF and EGF and increasing B27 concentration 10 times (Falk et al., 2012; Koch et al., 2009). We assigned the name of NES differentiation medium to this medium (see Table 6). As it was indicated above for NES cell culture section, it is also necessary to pre-warm NES differentiation medium.

Day 0

69. Culture NES cells according to the above guidelines.
70. Seed NES cells in NES growth media with a density of 40 000 cells/cm^2^.

Day 1

71. Remove NES growth medium by slightly tilting the culture plate and replace it with an equal volume of NES differentiation medium (see Table 1).

Day 2–7

72. Replace half of NES differentiation medium every other day without tilting the plate.

Day 7 until intended day of differentiation

73. Carefully replace half of NES differentiation medium every other day.
74. Add mouse laminin (1:500) to the media for every feeding

**CRITICAL:** Do not induce neuronal differentiation right after NES cells thawing. Culture the cells for at least 24h in NES medium before changing the medium to differentiation medium.

**CRITICAL:** NES cells under differentiation and eventually neurons only loosely attach to the growth surface. We therefore recommend being extra careful when handling plates, i.e., while moving plates in and out of the incubator or changing medium. Additionally, we recommend using a p1000 pipette for the removal of media, instead of aspiration with a vacuum pump.

**CRITICAL:** Doubling the amount of NES differentiation medium after two weeks of differentiation can be beneficial.

**CRITICAL:** To document neuronal differentiation morphology, bright-field imaging represents an informative and quick procedure (Figure 1). Differentiating cells should not be removed from the incubator for extended periods of time and we therefore recommend placing cell incubator, cell hoods, and microscope close to one another to minimize neurons detachment during transport.

**CRITICAL:** We recommend supplementing the culture plate with fresh mouse laminin (1:500 final concentration) for every feeding during differentiation starting from day 7 to prevent detachment of the neurons.

#### Modulation of neuronal differentiation through redox homeostasis

**Timing: 1 h**

The growth conditions mentioned above are sufficient for NES cell culturing and differentiation. One should be aware that redox homeostasis is tightly coupled to stem cell differentiation and function (Li et al., 2019 and Nugud et al., 2018). We here present consequences on NES cell differentiation, when antioxidant levels differ from those recommended in the standard NES differentiation medium mentioned above.

The following protocol compares differentiation potential using standard B27 (as above) or B27 minus antioxidants (B27-MA) in the NES cell differentiation medium in the presence or absence of the antioxidant N-Acetyl-L-cysteine (NAC). B27-MA is lacking antioxidant power: vitamin E, vitamin E acetate, superoxide dismutase, catalase, and glutathione. Thus, we can study how the lack of antioxidant supplementation in the medium is affecting neuronal differentiation.

75. Follow NES cells establishment guidelines for correct storage of factors and medium. B27-MA is stored and aliquoted as B27.
76. Prepare NES differentiation medium with B27 or B27-MA (see Table 7).
77. Prepare 6 wells to evaluate the effect of antioxidants on neuronal differentiation:
  a. B27 no NAC
  b. B27 + 0.5 mM NAC
  c. B27 + 2.5 mM NAC
  d. B27-MA no NAC
  e. B27-MA + 0.5 mM NAC
  f. B27-MA + 2.5 mM NAC

**Timing: 30 min**

Culture for at least 14 days for a completely developed neuronal morphology.

Day 1

78. Plate the cells as mentioned in the NES cell differentiation part. Remove NES growth medium 24 h after seeding of the cells by slightly tilting the culture plate and replace it with an equal volume of the 6 different NES differentiation mediums mentioned above.

Day 2–14

79. Replace half of NES differentiation medium every day.
80. Add mouse laminin to the media every time the media gets changed.

**CRITICAL:** This experiment needs daily attention and documentation since cells are extremely sensitive to a deregulation in ROS levels and can quickly die.

**CRITICAL:** As in the normal differentiation handling of the plates and adding media must be performed carefully. Add fresh media to the side of the plate wall as slow as possible.

**CRITICAL:** Remove the media carefully when changing the media.

### Nucleofection of NES cells

**Timing: 1 h**

We noticed extensive NES cell death after transfecting NES cells with Lipofectamine 3000 (Thermo Fisher Scientific) and therefore explored nucleofection as transfection method.

81. Collect 2.5 × 10^5^ NES cells for one well in a 16-strip and 2 × 10^6^ for one cuvette according to NES cell culture protocol above but pellet cells by slow centrifugation at 80 *g* for 10 min.
82. Resuspend cells in 20 μL of pre-warmed P3 buffer for one strip well and 100 μL for one cuvette.
83. Add 0.6 μg and 5 μg of the desired DNA for the strips and cuvettes, respectively.
84. Transfer the NES cells-DNA mix into the strip or the cuvette.
85. Place the strips or cuvette into the nucleofector and run program DC100 (this is the most efficient program to nucleofect NES cells).
86. After nucleofection let the cells recover for 10 min at 21°C to reduce cell death. Gently transfer the transformed cells to NES growth medium and seed them in a coated 24 multi-well plate (strip) or T25 flask (cuvette). Add ROCK inhibitor (1:1000) for better survival of the cells.
87. After 24 h, change medium according to NES cell culture conditions (see Table 1 (volume) and Table 5 (media composition)).
88. For stable line generation, antibiotics can be incorporated into the medium after 48 h. For transient experiments the peak of expression is reached at 48–72 h.

**CRITICAL:** Avoid making bubbles when placing cells in the strip or the cuvette.

**CRITICAL:** For stable cell lines generation, the antibiotic selection should be added after 48–72 h.

**CRITICAL:** Make sure to never culture NES cells in densities lower than stated in the above sections.

## Expected outcomes

Albeit sensitive to culture conditions, NES cells are highly proliferative and can provide an almost unlimited source of cells of a neuronal cell lineage.

To verify the cell lineage, check for expression of the NES cell markers SOX2 and NESTIN by immunohistochemistry, FACS or qPCR. Additionally, the newly established NES cell lines should present the typical rosette shape (Figure 1).

**Figure 2:**
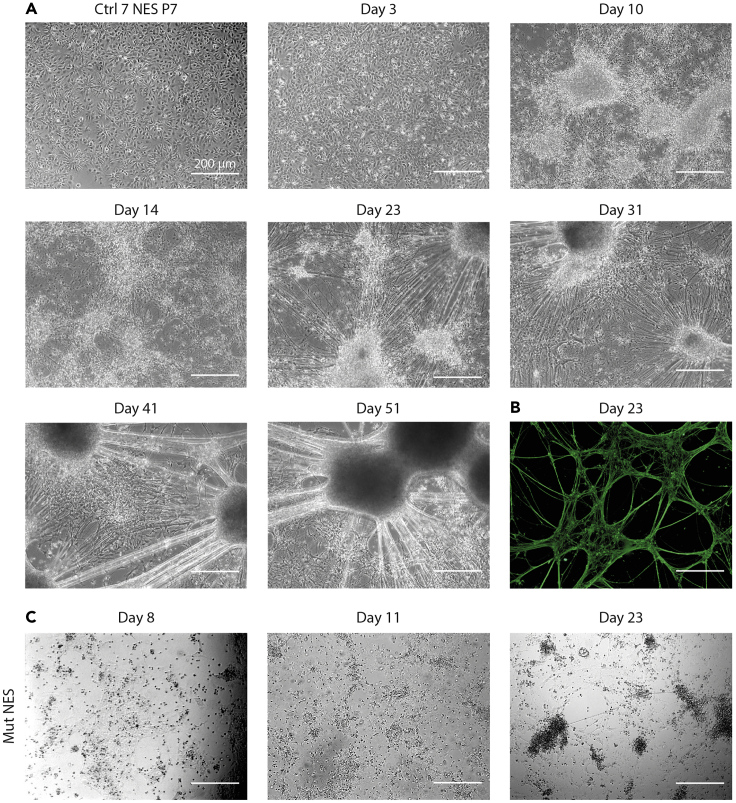
NES cells can efficiently differentiate into neural cells (A) Representative bright-field image of a healthy control NES cell line differentiated for 51 days. After onset of differentiation, NES cells progressively assume a more branched morphology with neurites growing out from the cell body. The more differentiation progresses the more the cell bodies cluster together. (B) To monitor neuronal differentiation in a healthy control cell line, cells can be immune-stained with B-III-Tubulin (green). (C) Example of a differentiation deficient mutant NES cell line (1). Scale bar: 200 μm.

During the first 4 days NES cells will arrest, after which (day 5–6) characteristic projections of neuronal morphology will appear. Cells will continue to differentiate and cluster in groups of cell bodies with bundled projections connecting the different clusters. After approximately 45 days of differentiation glial cells are formed. Representative images of neural differentiation are shown in Figure 2A.

The outcome of differentiation can be tested by immunohistochemistry e.g., with the neuronal marker β-III tubulin (Figure 2B). Other neuronal markers might also be of use, but we recommend using β-III tubulin for a quick determination of the quality of differentiation. We also recommend monitoring the decline of stem cell markers, such as SOX2, during differentiation, since mature neurons should present very low levels in comparison to NES cells. After approximately 45 days of differentiation astrocytes expressing typical glial markers can be detected in the differentiating cells.

A failed differentiation attempt can be seen in Figure 2C.

**Figure 3:**
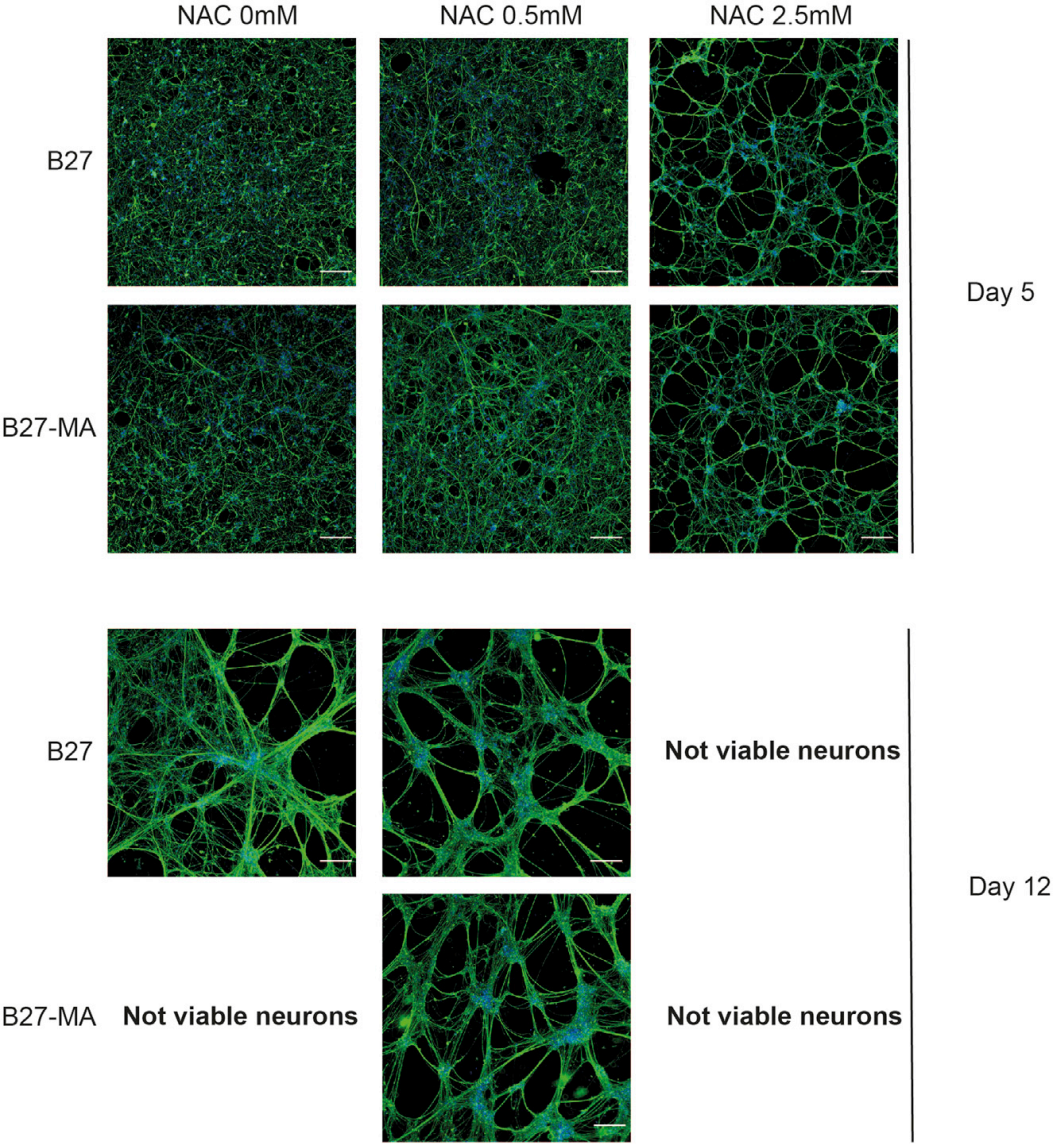
Neuronal differentiation potential in standard NES cell differentiation media or media with high or low antioxidant capacity Representative B-III-Tubulin staining (green) of NES cells at different points of differentiation (day 5 and day 12). Differentiation was started with either standard B27 (B27), or with B27 deprived of its antioxidant components (B27-MA). Moreover, differentiation medium was supplemented with either 0.5 mM or 2.5 mM N-Acetyl Cysteine (NAC), a commonly used antioxidant molecule. The complete removal of antioxidant power from the B27 results in non-viable differentiated cells. Addition of 0.5 mM NAC efficiently rescues cell death, but an increase of NAC concentration to 2.5 mM eventually leads to complete loss of the culture, suggesting that a precise balance between oxidant and antioxidant actions is required for normal differentiation. Scale bar: 100 μm.

Neuronal differentiation is blocked by the absence of antioxidants in NES differentiation medium with B27-MA at day 11 followed by neuronal cell death at day 12. NES differentiation medium with B27-MA supplemented with low NAC concentrations (0.5 mM) rescues this block allowing neurons to completely develop. High concentration NAC (2.5 mM) supplemented NES differentiation medium speeds up neuronal differentiation but failed to keep neurons alive inducing neuronal cell death at day 7 (Figure 3).

## Limitations

NES cells present a few limitations that are intrinsic to the model. Their stemness properties result in a broad spectrum of differentiation outcomes, eventually leading to a mixed culture of different subtypes of neurons and glia. It is possible to direct differentiation to a neuronal subtype of interest using alternative differentiation protocols, but we have not presented this here.

Moreover, it is important to use standardized culturing conditions, such as 5% CO_2_ and 21% O_2_, in order to have comparable cell models. If NES cells are not cultured under such conditions variability among cell lines can be introduced. The use of multiple lines, for both control and patient cell lines, is recommended to ensure reproducibility.

It should be considered that the isolation and culturing of NES cells might lead to clonal selection during the process due to the culturing conditions used, like cell density. It is therefore recommended to use the culture conditions discussed in this protocol. Always check the quality of your NES cell cultures before they are used in experiments.

The NES cell system is reductionistic, thus, it might be difficult to study interactions with other cell types similar to the in vivo situation. NES cells are generated from iPS cells and the reprogramming process to establish the iPS cells includes erasing the epigenetic landscape. The derived NES cells might not display the same epigenetic markers present in the individual donated cells for reprogramming.

It is difficult to study complex cell-cell interaction with other cell types as well as the impact of complex environmental factors, since these cells are kept in a monolayer culture.

This protocol describes 2D culture of NES cells. If the interactions of different neural cell types should be studied in 3D we recommend using protocols for brain organoids.

Using NES cells and differentiated cells in a 2D culture comes with the limitation of not knowing how and if a specific genotype translates into a specific behavior. For investigations regarding research like this a combined in vitro in vivo research approach is necessary.

## Troubleshooting

### Problem 1

NES cells detach from the dish (step 41–44)

### Potential solution

Residual Poly-L-Ornithine or the absence of mouse laminin will prevent NES cells from attaching to the growth surface. We recommend at least three washes after Poly-L-Ornithine coating with PBS. A mouse laminin concentration of 2 μg/mL was only partially sufficient to maintain NES cell growth. We therefore suggest a 4 μg/mL concentration, which significantly improved NES cell growth. In addition, while preparing culture plates for NES cell culture, 12 h incubation at +4°C with mouse laminin showed better results in comparison to 2 h at 37°C. We also recommend adding fresh laminin (4 μg/mL concentration) for every feeding staring from day 7 of NES cell differentiation. Make sure that an appropriate culture dish is used (see key resource table).

### Problem 2

Excessive cell death after trypsinization or during splitting (step 41–44)

### Potential solution

Pre-warm TrypLE select, wash medium and NES growth medium and avoid excessive pipetting of NES cells. Never trypsinize the cells for more than 3 min and proceed with the splitting procedure as quick as possible so the cells don't have to stay outside of a culture plate for longer period of times. Aspirate laminin coating right before seeding the cells, and do not let the dish dry out. Double check time and speed of centrifugation.

### Problem 3

NES cells remain quiescent (step 71–74)

### Potential solution

Double check factors concentration and the dilution used to prepare the aliquots. Do not store the factors at +4°C. Use fresh bFGF, EGF and B27 to complete NES growth medium to improve factors activity. Avoid constant opening and closing of the incubator so the temperature and CO_2_ concentrations remain constant. It might also help to change only half of the media for every media change until the cells recovered. Check for the right seeding density. Seeding at a too low density can influence the cells proliferation rate.

### Problem 4

Excessive cell death during neuronal differentiation (step 71–74)

### Potential solution

Low cellular density can induce a quiescent state and cell death in NES cells. To achieve a successful neural differentiation, the confluences stated in this protocol should be used. Aberrant differentiation and high rates of cell death are expected below that percentage. In the beginning of the differentiation process cell death to some extend is expected.

### Problem 5

Excessive cell death during nucleofection (step 81–88)

### Potential solution

Increasing the number of cells and/or decreasing the amount of DNA used can reduce cell death. Avoid excessive pipetting and pre-warm NES growth medium. Adding ROCK inhibitor to the media before and after the nucleofection can help the survival rate as well.

### Problem 6

Pelleting of NES cells after harvesting with a scraper (step 60–63)

### Potential solution

Alternative to TrypLE trypsinization, cells can be harvested using a cell scraper. We recommend taking extra care as cells will readily detach. Preferably, harvest in NES growth medium followed by a PBS wash and a new centrifugation at 300 *g* for 3 min.

## Resource availability

### Lead contact

Further information and requests for resources and reagents should be directed to and will be fulfilled by the lead contact, Anna Wredenberg MD, PhD, anna.wredenberg@ki.se

### Material availability

This study did not generate new unique reagents.

### Data and code availability

This study did not generate/analyze [datasets/code].
